# Secondary Air Pollutants and Forests — New Perspectives

**DOI:** 10.1100/tsw.2007.18

**Published:** 2007-03-21

**Authors:** J. Neil Cape

**Affiliations:** Centre for Ecology and Hydrology, Bush Estate, Penicuik, Midlothian, EH26 0QB, UK

**Keywords:** ozone, photochemical oxidants, fine particulate, plant effects, pollutant interactions

## Abstract

Air pollution has been known to affect forests for over a century, and many of the mechanisms of pollutant deposition and effects have been established, at least for forest trees. Changes in air quality as a result of emission controls in Europe and North America, or as a result of rapid industrialisation in southern and eastern Asia, have highlighted new pollution problems. This paper, by reference to recent publications, highlights two areas where more research is required: the interactions of photochemical oxidants with biogenic emissions of volatile organic compounds, and their impact on ecological signalling; and the role of atmospheric particles in changing the leaf surface environments in forests.

## INTRODUCTION

The effects of air pollution on forests have been studied for well over a century, as evidence of the impact of smoke and other emissions from industrial activity on trees close to chimneys was reported and investigated. Pollutants from coal burning (initially identified as “smoke” and sulphur dioxide [$SO_2$]) were seen as the major culprits, and attempts were made to map out the spatial and temporal extent of these harmful effects[1,2]. More recently, the political discussion in Europe over “Forest Decline” (Waldsterben) during the 1980s extended the debate to cover the long-range transport of pollutants, and the role of secondary pollution as “acid rain” in acidifying soils or leaching the base cations necessary for healthy tree growth. The establishment of a systematic forest monitoring programme in Europe, along with measures of air quality and precipitation chemistry, has done much to show the relative importance of pollution, climate, and disease on the apparent health of forest trees (www.icp-forests.org). The introduction of Critical Levels and Critical Loads[3] as a means of advising the control of pollutant emissions, along with changes in fuel use from coal and oil to gas, has led to greatly decreased severity of “acute” exposure of the environment to high concentrations of airborne pollutants. However, the chronic effects and the legacy of a century of acid deposition on European soils will take longer to address.

Although Europe and North America are now attempting to redress the excesses of the past century, other parts of the world, in particular India and China, are rapidly developing their economies and their dependence on coal combustion as the major source of energy is inevitably leading to greatly reduced air quality and the potential for serious environmental effects on forests. Although the acute problems of direct exposure to smoke and/or $SO_2$ are similar to those experienced in Europe and North America, but with different species involved and different climatic regimes, the particular problems of soil acidification may be less of a threat given the availability of base cations in soil and the input of base cations in dust storms over much of the region. However, the long-term capacity of soils to neutralise acid deposition at the levels now experienced remains to be seen. Moreover, the rapid expansion of the human population, and of agricultural developments (including animal numbers) to meet the need for food, has led to a large increase in emissions of ammonia gas. While this may act to prevent the formation of acidic aerosol particles, the deposited ammonium will increase soil acidity, and the ammonium sulphate and nitrate aerosols formed add to emissions of "smoke" (black carbon and mineral dust) to create regional haze. It has been estimated that, even in the absence of direct air pollution effects on plants, the loss of photosynthetically active radiation caused by the haze might be responsible for losses of up to 30% of crop yields in China[4]. Presumably, similar effects would be observed for forests, even though, as in Europe and North America, many will benefit from the added nitrogen from wet and dry deposition, in terms of timber production[5].

The increasing emissions of pollutants from the combustion of fossil fuels, whether in power stations or vehicles, is leading to a global increase in the production of tropospheric ozone, especially in the northern hemisphere[6,7]. Estimates of the intercontinental transport of ozone and/or its precursors have been made and indicate the extent to which the control of photochemical oxidants is within the control of individual countries or regions[8,9,10,11]. In Europe and North America, emission controls on precursors have led to a reduction in the severity of ozone "episodes", as measured by the peak concentrations[12,13], but concern has been expressed at the apparently inexorable increase in background concentrations to concentrations where direct effects on vegetation are likely to occur[14]. It is also becoming apparent that increasing emissions of ammonia gas from intensive agriculture (animal husbandry)[15] are leading to the production of ammonium sulphate and nitrate particles[16], which are only slowly removed from the atmosphere. Little is known about potential direct effects of airborne particles on vegetation, although indirect effects of light attenuation and possibly increased cloudiness and consequent changes in rainfall patterns have been recognised, as noted above.

Given the above global changes in the patterns of exposure of forests to air pollutants, what are the scientific challenges to an understanding of the potential impacts? This short article cannot provide a comprehensive review of the accumulated knowledge of the effects of air pollutants on forests, but draws attention to two areas for potential future research, where increasing levels of air pollution may be causing hitherto unrecognised effects. These are (1) the interactions of ozone and its manmade precursors, volatile organic compounds (VOCs) and nitrogen oxides (NOx), with biogenic emissions of volatile organic compounds; and (2) the effects of ammonium salts on vegetation. Although the discussion is not specific to forests, trees are major emitters of biogenic VOCs and efficiently intercept small particles from the atmosphere. Moreover, research on forest effects to date has often focussed on trees rather than forests, yet it may be the effects of pollutants on the interactions of trees with other forest plants, fauna, parasites, pathogens, and the underlying soil that determine the long-term effects on forests.

## OZONE AND BIOGENIC VOCS

The photochemical production of ozone in the troposphere relies on the presence of two key precursors: VOCs and NOx. In industrialised and densely populated regions where NOx emissions are large, the biogenic contribution of VOCs may provide the necessary precursors for ozone production[17]. Although this has long been recognised, the ecological and physiological roles of these biogenic VOCs have not been well established[18]. Release of such energy-rich molecules into the atmosphere must have evolved as an important control mechanism, perhaps as a means of dissipating absorbed sunlight that cannot be used for photosynthesis of sugars, thereby avoiding the buildup of reactive oxidant species (ROS) inside leaf cells. The molecules involved may also act as signals of herbivory[19], triggering defence responses in nearby plants[20] or attracting predators[21]. Emissions of monoterpenes and other VOCs increase in response to herbivory, for example, ocimene and linalool (monoterpenes) and farnesene (sesquiterpene)

from crop plants[22], and α- and β-pinene and δ-3-carene from conifer needles[23]. Such complex ecological responses have evolved in an atmosphere with markedly less ozone than at present — how might increased ozone levels interfere with such signalling roles, or even the signalling roles attributed to ROS within leaves[24,25]?

Exposure of leaves to enhanced ozone concentrations also leads to increased emissions of VOCs[26], presumably because the type of injury induced provokes the induction of repair and response mechanisms in the leaf that mimic those that have evolved to respond to insect attacks[27]. Biogenic VOCs are typically reactive to ozone, containing carbon-carbon double bonds; examples include ethene[28] and isoprene[29], but also include much more complex molecules such as E-(β)-farnesene that have multiple double bonds (susceptible to rapid oxidation), and are known to attract predators of herbivorous insects[30]. The potential, therefore, arises not only for stimulation of defensive responses (in terms of VOC emissions), but also the degradation of signalling pathways because of the rapid destruction of signalling molecules by ozone, or more likely by the hydroxyl radical that is part of the complex oxidising mixture formed during photochemical ozone formation[31].

Biogenic VOCs from forests are also involved in production of secondary organic aerosols (SOA), the fine particulates that form the blue haze associated with forests even in unpolluted conditions. These particles are formed from the oxidation of biogenic VOCs[32], as the complex organic molecules are oxidised to form more polar compounds that can agglomerate, or attach themselves to pre-existing inorganic dust particles. As oxidation proceeds with the production of polar moieties (alcohols, carbonyls, carboxylic acids) these organic-coated aerosols change from being hydrophobic to hydrophilic[33], absorb atmospheric water and grow (eventually) into droplets. Increasing oxidation rates mean that this process will occur more rapidly, leading to enhanced rates of particle and droplet formation. Such an effect is likely to be exacerbated in the presence of higher concentrations of fine inorganic particulates (see below) on which the oxidised VOCs can accumulate.

## FORESTS AND FINE PARTICULATE MATTER

SOAs are only one component of the particles in the atmosphere. Industrial and vehicular emissions of acidic compounds ($SO_2$ and NOx) react in the atmosphere with ammonia to form particles that can be transported long distances before eventually being incorporated into precipitating clouds, or being transferred by dry deposition to the earth's surface. The latter process is relatively inefficient, particularly for short vegetation[34], so atmospheric lifetimes of fine particulate matter (diameter $<10^{-6}$ m) may be several days. Deposition rates of fine particles to forests are relatively rapid, although still small compared to deposition rates for the precursor gases. However, the increasing concentrations of fine particles in air, particularly in regions with rapidly developing economies, will lead to increasing deposition of particles to forest surfaces. What are the potential impacts of such deposition? In the worst case, close to major industrial sources of solid particles (e.g., fly ash), leaf surfaces may be coated with water-insoluble material, interfering with light absorption or even blocking open stomata and affecting water regulation[35].

On a wider scale, increasing deposition of water-soluble particles, such as ammonium salts, will lead to an increase in the frequency of surface wetness on leaves. Ammonium salts are hygroscopic, absorbing water from the atmosphere when relative humidity exceeds 70% (for the case of pure ammonium sulphate)[36]. This means that a surface on which ammonium salts have been deposited will be "wet", at least in terms of a thin film of moisture, even in the absence of rain, provided that relative humidity is high enough. The proximity of the leaf surface to a source of water vapour from open stomata, and locally enhanced humidity, may lead to conditions of surface moisture even when atmospheric humidity within the canopy is lower than the theoretical hygroscopic limit, and may lead to enhanced water loss from canopies[37] and enhanced deposition rates of water-soluble pollutant gases[38]. The effect is similar to the formation of dew, which is already recognised as important in regulating the exposure of leaf surfaces to air pollutants[39,40,41]. Surface wetness also increases the possibility of ion exchange processes, leading to enhanced leaching of base cations from forest canopies. A systematic increase in leaf surface humidity, and the availability of nutrient N, is also likely to encourage the proliferation of epiphytic flora and increase the likelihood of pathogen infection[42]. Certainly, the proliferation of algae on leaf surfaces of conifers in the Netherlands has been reported in response to increased air concentrations of ammonia[43], but this extreme example may be repeated albeit to a lesser extent elsewhere. Such subtle changes in the environment of the phylloplane are not easily amenable to direct experimentation, so little is known quantitatively of the potential risk posed by such processes. The complex biological processing of nutrients on leaf surfaces is shown, for example, by the production and consumption of both inorganic and organic N, and consequent changes in throughfall chemistry[44,45], but detailed understanding of these processes is lacking, even in the absence of air pollutant interactions.

## CONCLUSIONS

Air pollution effects on trees and, by extension, on forests has been recognised for over a century, yet the patterns of pollution, both geographical and chemical, are constantly changing. Although some success has been achieved in reducing acute pollution locally and regionally by the use of cleaner fuels and controls on emissions, the rapid development of fossil fuel combustion in Asia, and the intensification of agriculture both there and in Europe and North America, is leading to an increasing potential for the long-range transport of air pollution problems. Airborne fine particulates are harmful to human health, and concerns have led to the imposition of target concentrations and regulatory powers in an attempt to control air concentrations. We do not know their importance to forests, where deposition of particulates may not lead to quantitatively large rates of nutrient deposition, yet may subtly change the properties of leaf surfaces and the environment in which phylloplane organisms live. Adding to this uncertainty is the role of forests themselves, in a more oxidising climate, of adding to the generation of SOAs, or even to the generation of photo-oxidants through enhanced VOC emissions. The recognition that subtle ecological interactions could be modified by photo-oxidants points to a role for air pollution in changing the way forest ecosystems function, even if there are no obvious direct effects on tree health.
